# Educational case: Estrogen-receptor positive breast cancer: Diagnosis, response to therapy, and prognosis

**DOI:** 10.1016/j.acpath.2025.100210

**Published:** 2025-08-12

**Authors:** Ahmed Afifi, Phoebe Hammer, Kelly Ernst

**Affiliations:** a Benha University Faculty of Medicine, Benha, Al-Qalyubia Governorate, Egypt; b Stanford University, Palo Alto, CA, USA

**Keywords:** Pathology competencies, Organ system pathology, Breast, Breast neoplasms, Morphologic classification, Prognostic and predictive factors, Gene expression profiling

The following fictional case is intended as a learning tool within the Pathology Competencies for Medical Education (PCME), a set of national standards for teaching pathology. These are divided into three basic competencies: Disease Mechanisms and Processes, Organ System Pathology, and Diagnostic Medicine and Therapeutic Pathology. For additional information, and a full list of learning objectives for all three competencies, see https://doi.org/10.1016/j.acpath.2023.100086.^1^

## Primary objective

Objective BR2.7: Factors Affecting Response and Prognosis of Breast Cancer. Explain the prognosis and likelihood of recurrence and response to therapy for patients having breast cancer based on knowledge of molecular classification and/or gene expression profiling, morphologic classification, grade, prognostic marker studies, and other predictive factors.

Competency 2: Organ System Pathology; Topic: Breast (BR); Learning Goal 2: Breast Neoplasms.

## Patient presentation

**Fig. 1:**
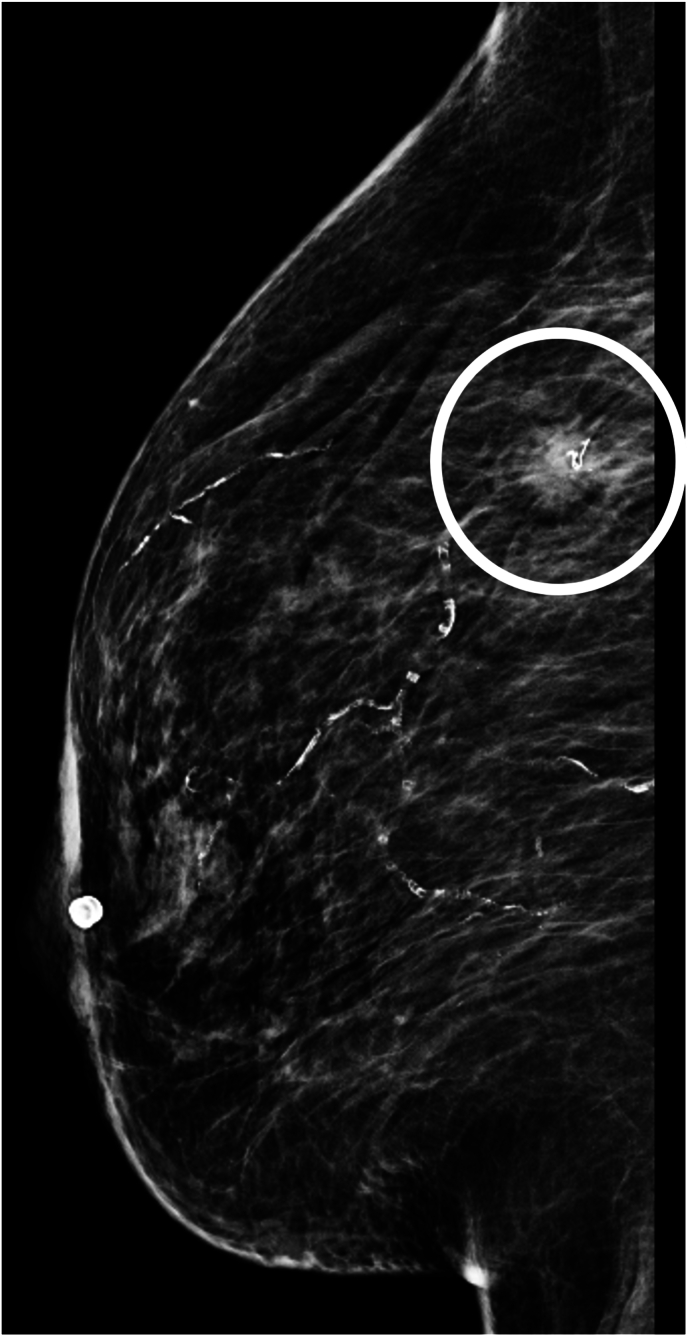
An approximately 2-cm spiculated mass was identified on routine mammography.

A 72-year-old woman with no significant past medical history, including prior normal screening mammograms since age 50 years (last mammogram 24 months prior), presents for routine mammogram. Screening identifies an approximately 2-cm spiculated mass in her right breast (Fig. 1).

## Diagnostic findings, Part 1

**Fig. 2:**
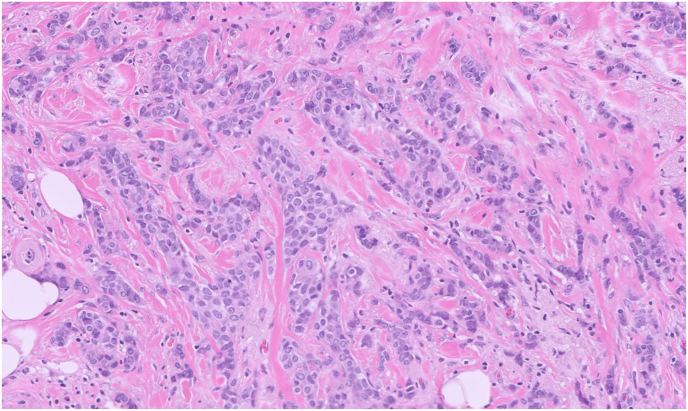
Core biopsy sections show carcinoma cells in clusters infiltrating stroma (20X).

The patient undergoes an ultrasound-guided core biopsy of the right breast mass with a representative histologic image, as shown in Fig. 2.

## Questions/discussion points, Part 1

### Describe the histologic findings in Fig. 2

Sections of the right breast mass show malignant epithelial cells infiltrating stroma in irregular nests. The malignant cells have ovoid nuclei, fine chromatin, variably prominent nucleoli, and delicate eosinophilic cytoplasm with indistinct cell borders.

### What is the diagnosis?

The patient is diagnosed with invasive ductal carcinoma (IDC), provisional grade 2 of 3 (intermediate grade) (Fig. 2). The degree of tubule formation, nuclear pleomorphism (variation in nuclear size and shape), and mitotic index determine the breast cancer grade. Morphologic classification of breast cancer is essential for understanding the different histologic subtypes, which can influence prognosis and treatment options. The most common subtypes include IDC, invasive lobular carcinoma, and ductal carcinoma *in situ*, each characterized by distinct cellular features and growth patterns that can impact clinical management.

### What biomarkers should be ordered on the biopsy specimen to assess newly diagnosed invasive breast cancer?

The standard biomarkers include estrogen receptor (ER), progesterone receptor (PR), human epidermal growth factor receptor 2 (HER2), and sometimes Ki-67, which is not routinely performed in all cases. These biomarkers offer valuable insights into the molecular subtype of the cancer and guide treatment decision-making.^2^^,^^3^

### What techniques are utilized to assess biomarkers, and what is their clinical importance?

Immunohistochemistry (IHC) is utilized to evaluate breast biomarkers on formalin-fixed paraffin-embedded tissue. Estrogen receptor and PR expression by IHC is used to determine the potential efficacy of hormone therapy. Human epidermal growth factor receptor 2 status is determined using various modalities including IHC with or without *in situ* hybridization (ISH) and determines the eligibility for HER2-targeted therapies. Ki-67 IHC is a marker of cell proliferation and is used to assess tumor aggressiveness. Clinically, a high Ki-67 index may suggest a more aggressive tumor, influencing the decision to pursue neoadjuvant chemotherapy, especially in cases where other biomarkers present an ambiguous prognosis.^4^ These biomarkers guide treatment decisions and provide valuable predictive and prognostic information.^5^

### What other factors impact breast cancer prognosis?

Other factors that impact breast cancer prognosis include patient age, status of several germline gene variants including *BRCA1* and *BRCA2*, overall health status, cancer stage at the time of diagnosis, tumor size, histologic subtype, histologic grade, and the presence of axillary lymph node involvement. Each of these factors contributes to the overall assessment of disease progression and predicts response to treatment, helping clinicians tailor personalized treatment plans for better patient outcomes.^6^, ^7^, ^8^

## Diagnostic findings, Part 2

**Fig. 3:**
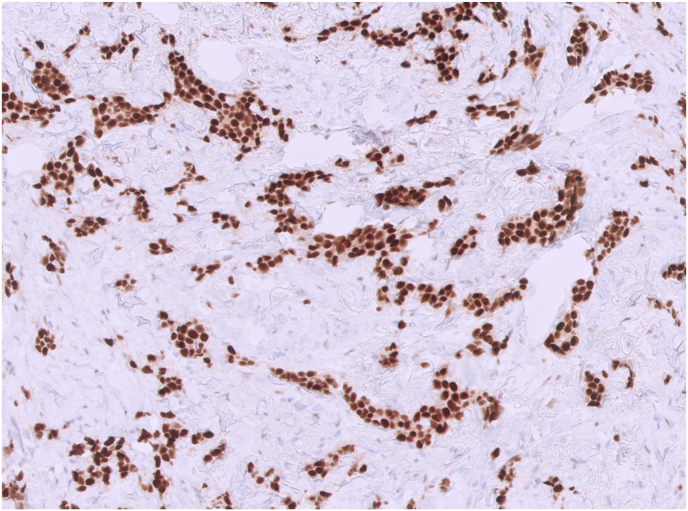
Strong tumor nuclear staining with estrogen receptor is identified on immunohistochemistry (20X).

Ancillary IHC for the aforementioned biomarkers is performed on the patient's core biopsy tissue. The carcinoma is positive for ER (Fig. 3) and negative for HER2.

## Questions/discussion points, Part 2

### What is the clinical significance of ER positivity in breast cancer?

The assessment of ER expression holds crucial clinical significance as it enables targeted hormonal therapy, such as selective ER modulators (e.g., tamoxifen) or aromatase inhibitors (e.g., anastrozole), which effectively reduce the estrogen signaling pathway and inhibit tumor growth. Determination of ER status in breast cancer patients guides treatment decisions and improves patient survival.^9^

### What are the clinical risk factors and epidemiologic features of ER-positive breast cancer?

Estrogen receptor–positive breast cancer is the most prevalent subtype, accounting for 70–80% of cases. The overall breast cancer incidence is about 86 cases per 100,000 women yearly, with ER-positive tumors representing the majority.^10^ It primarily affects postmenopausal women, with an increased risk associated with early menarche and late menopause.^11^ The lifetime risk of breast cancer is approximately 13%, varying with hormonal and genetic factors.^12^

Hormonal factors play a significant role in the development of breast cancer. Lifetime exposure to estrogen influences its occurrence, and certain factors such as hormone replacement therapy and obesity have been linked to a higher risk. Additionally, a family history of breast cancer and specific genetic mutations contribute to an individual's susceptibility to the disease.^13^ Understanding clinical and epidemiologic factors is crucial for effective breast cancer prevention and management.

### What is the prognosis of low-grade ER-positive HER2-negative breast cancer?

This type of ER-positive breast cancer typically has a more favorable prognosis than ER-negative breast cancer due to its responsiveness to hormonal therapies. Research has indicated that patients diagnosed with ER-positive breast cancer have a lower risk of recurrence and higher overall survival rates than those with ER-negative breast cancer. Specifically, for early-stage ER-positive cancers, five years of adjuvant (post-surgical excision) therapy with tamoxifen resulted in a 34% reduction in the annual death rate and 9% reduction in absolute mortality at 15 years.^14^ While ER-positivity generally indicates a relatively better prognosis, many factors influence patient prognosis and outcomes including the stage and grade of the cancer, the patient's age and overall health, and the efficacy of the treatment administered.^13^ Stage remains the most important prognostic factor in breast cancer with five-year disease-free survival rates exceeding 95% in patients with stage I disease, which decreased to 85% when regional lymph nodes are involved and to about 25% with metastatic disease.^15^ High histologic grade, large tumor size, and positive margin status are other pathologic features associated with worse outcomes.^15^ The lack of *HER2* expression is also independently associated with improved outcomes. *HER2* amplification leads to a more aggressive cancer that shows further overaction of pro-oncogenic pathways of unregulated cell growth. Although *HER2* expression is primarily used as a predictive marker for responding to treatment targeting the *HER2* pathway, *HER2*-positive patients still have significantly shorter disease-free intervals and poorer clinical outcomes.^13^ Recently, tumors with any HER2 IHC staining without *HER2* amplification by ISH (HER2-low breast cancer) have been shown to be associated with markers of better prognosis such as a low histologic grade.^16^

### Discuss the management of ER-positive breast cancer

The management includes a combination of surgery, radiation therapy, and systemic treatments such as hormonal therapy, standard chemotherapy, and targeted therapy. For patients with ER-positive, HER2-negative breast cancer, lumpectomy or mastectomy with adjuvant hormonal therapy is the mainstay of treatment. Tamoxifen is commonly used as adjuvant therapy in premenopausal women, while aromatase inhibitors are preferred in postmenopausal women. Furthermore, the duration of hormonal therapy may vary depending on individual risk factors and specific disease characteristics. The integration of adjuvant endocrine therapy with or without chemotherapy is considered based on the presence of positive and/or negative prognostic factors. Positive prognostic factors include ER-positive status, which is associated with better outcomes due to ER-targeted hormonal therapies, a low histologic grade, and a small tumor size, which are generally linked to a lower risk of recurrence. Additionally, a negative HER2 status, a low Ki-67 index, the absence of lymphovascular invasion, older age, and postmenopausal status are favorable indicators. On the other hand, negative prognostic factors include HER2-positive status, which is associated with more aggressive cancer, a high histologic grade, and a large tumor size, which increase recurrence risk. Positive lymphovascular invasion, a high Ki-67 index, multifocality, prior cancer history, and inheritance of germline gene variants such as *BRCA1* and *BRCA2* also contribute to a poorer prognosis. Tailoring treatment based on ER status helps to achieve more personalized and effective therapeutic approaches for ER-positive breast cancer patients. Patients may also be candidates for clinical trials of new endocrine therapies.^17^

## Diagnostic findings, Part 3

**Fig. 4:**
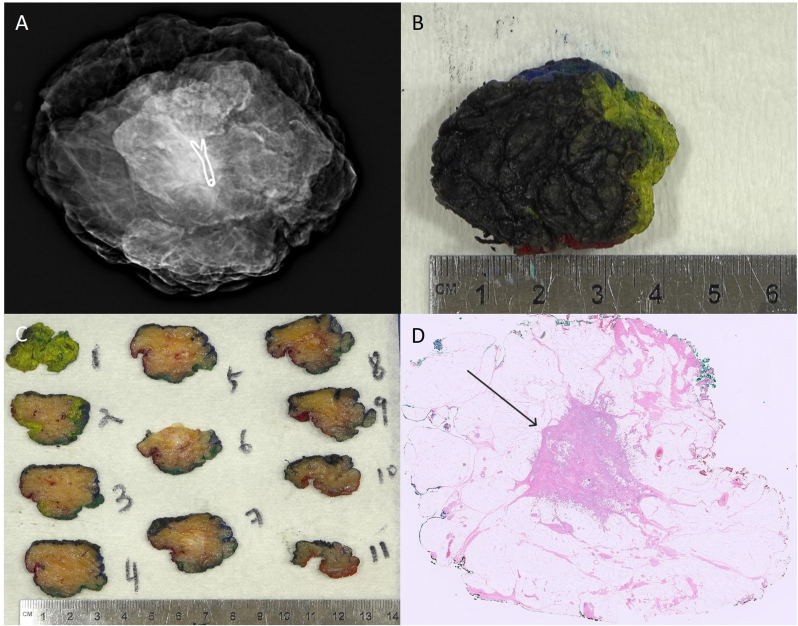
Radiologic (A), gross (B, C), and histologic (D, at 0.25X) photos of the sectioned lumpectomy are shown. The black arrow indicates the carcinoma (D).

The patient undergoes lumpectomy and sentinel lymph node excision. The final size of the tumor is 2.1 cm, correlating with imaging as seen on surgical specimen radiographs and serial sections of the excised tumor (Fig. 4). The surgical margins are negative for carcinoma, and the sentinel lymph node is negative for tumor. A paraffin-embedded tissue block is submitted for gene expression profile testing.

## Questions/discussion points, Part 3

### What is the process and key factors involved in breast cancer pathologic staging?

Staging breast cancer involves assessing the extent of growth and spread of the cancer. During macroscopic, “grossing” evaluation of the lumpectomy specimen, the tumor is measured and adequately sampled for subsequent histology measurement. Additionally, lymph nodes are serially sectioned and evaluated for metastasis. Patholoy staging parameters per the American Joint Commission on Cancer manual (AJCC 8^th^ edition) include tumor size, lymph node involvement, and metastasis.^18^^,^^19^ This patient has a tumor size of 2.1 cm, with no lymph node metastases and no known distant metastases, so she is assigned a pathologic stage of pT2 pN0(sn) pM0. The sn in parentheses denotes that only a sentinel lymph node evaluation was performed.

### How does the breast cancer stage impact the initial management plan?

The clinical stage of breast cancer dictates treatment options based on cancer severity and the likelihood of metastasis. For example, patients with early-stage breast cancer might be suitable candidates for breast-conserving surgery like lumpectomy (as seen in this case), while those presenting with more advanced disease may necessitate more aggressive treatments, such mastectomy with neoadjuvant or adjuvant chemotherapy, and adjuvant radiation therapy. Cancer stage also influences treatment timing and pre-treatment and post-treatment monitoring.^20^

### How does gene expression profiling influence the choice of systemic therapy in breast cancer patients?

Gene expression profiling assesses gene activity in tumors and can estimate recurrence risk. This testing helps determine if additional systemic therapies, like chemotherapy, are needed alongside endocrine therapy, allowing for personalized treatment plans. Oncotype DX® is a brand-name 21-gene assay preferred by the National Comprehensive Cancer Network Breast Cancer Panel for prognosis and prediction of chemotherapy benefit particularly for lower-stage hormone-receptor–positive HER2-negative tumors like that seen in this case.^20^^,^^21^ Alternative gene expression profiling tests, in addition to Oncotype DX®, may be used in various clinical contexts.

### What are the four main subtypes of breast cancer as defined by molecular grouping?

The four main types are luminal A (ER positive and HER2 negative), luminal B (ER positive and HER2 positive), triple negative (ER negative, PR negative, and HER2 negative), and HER2 enriched. Overall, cases that are ER positive, particularly luminal A and luminal B subtypes, tend to have a more favorable prognosis than triple-negative and HER2-enriched subtypes.^22^ This classification underscores the importance of molecular profiling in guiding treatment decisions.

### What are the challenges and potential mechanisms of resistance to hormonal therapy in ER-positive breast cancer?

**Fig. 5:**
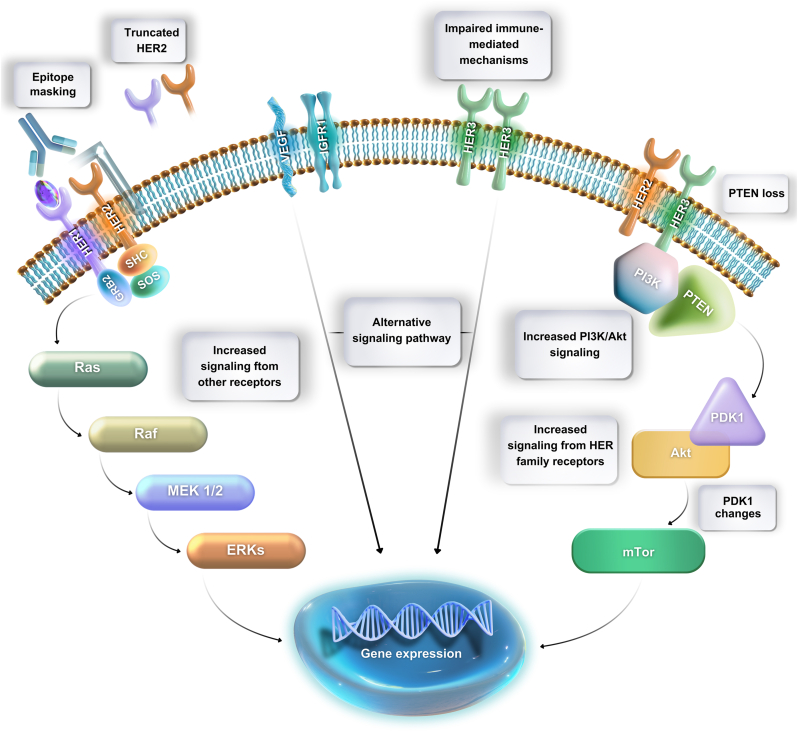
Relevant mechanisms of resistance to hormonal therapy in breast cancer are summarized.

Despite the effectiveness of hormonal therapy in ER-positive breast cancer, resistance to treatment can develop, leading to disease progression and treatment failure. Numerous resistance mechanisms have been identified (Fig. 5). One of the main challenges is the development of variants or alterations in the ER gene, leading to constitutive activation of the ER signaling pathway, even in the absence of estrogen.^23^

Additionally, the activation of alternative signaling pathways, such as the PI3K-AKT-mTOR pathway, can bypass the reliance on ER signaling and thereby evade the effects of hormonal therapies. Epigenetic changes and alterations in the tumor microenvironment can also play a significant role in promoting treatment resistance.^23^^,^^24^

Understanding the underlying mechanisms of resistance is vital to overcoming treatment failures in ER-positive breast cancer, enabling the development of effective strategies for improved outcomes. Combining hormonal therapies with targeted agents that inhibit the alternative signaling pathways or the use of combination therapies can potentially enhance treatment response and prevent or delay the development of resistance.^25^

## Teaching points

• Factors impacting breast cancer prognosis include patient age, overall health, tumor stage, tumor size, lymph node involvement, histologic grade, and the presence of certain gene mutations (e.g., *BRCA1* and *BRCA2*).
• Breast cancer staging involves tumor size, lymph node involvement, and metastasis.
• Clinical stage impacts the initial management plan by determining treatment options, timing, additional testing, and monitoring.
• Biomarkers evaluated in newly diagnosed invasive breast cancer include hormone receptor status (ERs and PRs), HER2 status, and Ki-67 expression.
• Biomarkers are clinically significant as they guide treatment decisions and provide information about prognosis.
• Estrogen receptor plays a crucial role in breast cancer development and stimulates cancer cell proliferation. Hormonal therapies like tamoxifen or aromatase inhibitors are used to selectively target the ER and effectively manage ER-positive breast cancer.
• Prognosis of ER-positive breast cancer is generally better than that of ER-negative breast cancer due to its responsiveness to hormonal therapies.
• Management of ER-positive breast cancer involves surgery, radiation therapy, hormonal therapy, chemotherapy, targeted therapy, and clinical trials.
• Hormonal therapy's efficacy in ER-positive breast cancer may be limited due to resistance mechanisms, including ER gene variants and activation of alternative signaling pathways like PI3K-AKT-mTOR. Understanding these mechanisms is vital for developing effective treatment strategies, such as combining hormonal therapies with targeted agents or combination therapies, to enhance treatment response and overcome resistance.

## Funding

The authors received no financial support for the research, authorship, and/or publication of this article.

## Declaration of competing interest

The author(s) declared no potential conflicts of interest with respect to the research, authorship, and/or publication of this article.
